# Synthesis and Sensory Evaluation of *ent*-Kaurane Diterpene Glycosides 

**DOI:** 10.3390/molecules17088908

**Published:** 2012-07-26

**Authors:** Indra Prakash, Mary Campbell, Rafael Ignacio San Miguel, Venkata Sai Prakash Chaturvedula

**Affiliations:** 1 Organic Chemistry Department, Global Research and Development, The Coca-Cola Company, One Coca-Cola Plaza, Atlanta, GA 30313, USA; 2 Product Development, Coca-Cola North America Division, The Coca-Cola Company, One Coca-Cola Plaza, Atlanta, GA 30313, USA; 3 Biosciences and Ingredient Department, Global Research and Development, The Coca-Cola Company, One Coca-Cola Plaza, Atlanta, GA 30313, USA

**Keywords:** *ent*-kaurane diterpene glycosides, catalytic hydrogenation, Pd(OH)_2_, structure characterization, spectral data, chemical studies, sensory evaluation

## Abstract

Catalytic hydrogenation of the three *ent*-kaurane diterpene glycosides isolated from *Stevia rebaudiana*, namely rubusoside, stevioside, and rebaudioside-A has been carried out using Pd(OH)_2_ and their corresponding dihydro derivatives have been isolated as the products. Synthesis of reduced steviol glycosides was performed using straightforward chemistry and their structures were characterized on the basis of 1D and 2D NMR spectral data and chemical studies. Also, we report herewith the sensory evaluation of all the reduced compounds against their corresponding original steviol glycosides and sucrose for the sweetness property of these molecules.

## 1. Introduction

**Figure 1:**
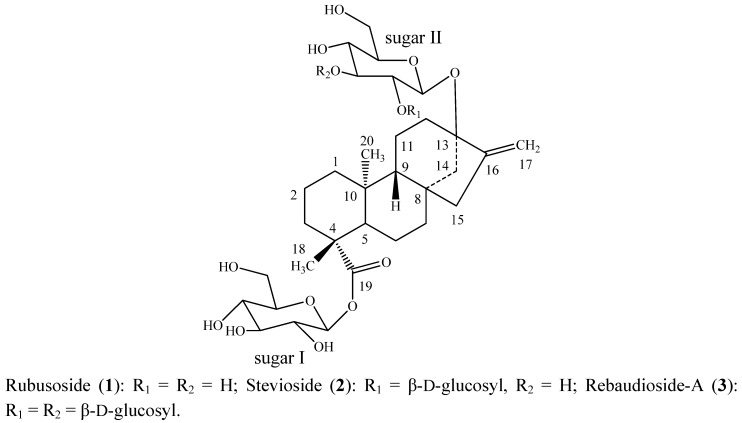
Structures of rubusoside (**1**), stevioside (**2**) and rebaudioside-A (**3**).

The major constituents isolated from the leaves of *Stevia rebaudiana* Bertoni (family: Asteraceae)are the potently sweet diterpenoid glycosides stevioside, and rebaudioside A. These compounds, which are known as Stevia sweeteners, are glycosides of the diterpene steviol, *ent*-13-hydroxykaur-16-en-19-oic acid [1] and are used to sweeten food products and beverages. Stevioside tastes about 150–250 times sweeter than sucrose, whereas rebaudioside A tastes about 200–300 times sweeter than sucrose and rubusoside taste about 100 times sweeter than sucrose; all are non-caloric. All three compounds—rubusoside (**1**), stevioside (**2**) and rebaudioside A (**3**)—have a β-D-glucosyl moiety at the C-19 position of the aglycone steviol as an ester, in addition to one β-D-glucosyl moiety at the C-13 position in **1**; a 2-substituted β-D-diglucosyl unit at the C-13 position in **2**, and a 2,3-substituted β-D-triglucosyl unit at the C-13 position in **3** (Figure 1). As a part of our continuing research to discover natural sweeteners, we have reported several glycosides from the commercial extract of *S. rebaudiana* [2,3,4,5,6,7,8,9].Apart from isolating novel compounds from *S. rebaudiana* and utilizing them as possible natural sweeteners or sweetness enhancers, we are also engaged in understanding the physicochemical profiles of steviol glycosides in various systems of interest and structural characterization of their metabolites as well as their synthesis [10,11,12,13]. In this article, we present the synthesis of novel *ent*-kaurane diterpene glycosides that are prepared by reduction of their C-16/C-17 exocyclic double bond; their structures were characterized on the basis of extensive NMR and MS spectroscopic data as well as enzymatic hydrolysis studies.

## 2. Results and Discussion

### 2.1. Chemistry and Sensory Studies

**Scheme 1:**
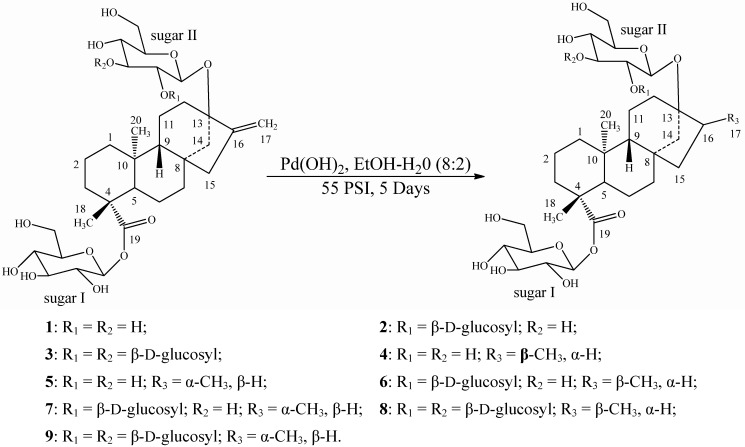
Hydrogenation of rubusoside (**1**), stevioside (**2**) and rebaudioside-A (**3**) and their reduced compounds.

Reductions of the three compounds rubusoside (**1**), stevioside (**2**), and rebaudioside-A (**3**) were performed using catalytic hydrogenation with Pd(OH)_2_ in a solvent mixture of EtOH/H_2_O (8:2) at room temperature under 55 psi H_2_ that furnished mixtures of dihydrorubusoside 1/2 (**4**/**5**), dihydrostevioside 1/2 (**6**/**7**), and dihydrebaudioside-A 1/2 (**8**/**9**), (Scheme 1), which are their corresponding 17α and 17β methyl group isomers. Further trials to separate the mixtures using various separation techniques failed; hence we are reporting these compounds as is.

The sensory evaluations of the synthetically reduced steviol glycosides at 500 ppm were performed against several control samples at 0.75%, 2%, 4%, 6%, and 7.0% sucrose equivalence (SE) in carbon treated (CT) water at room temperature (rt). Also, the sensory comparison of the mixtures **4**/**5**, **6**/**7**, and **8**/**9** against their original steviol glycosides was studied at 500 ppm using the controlled, multi-sip and swallow taste method as described in the Experimental. Results indicated that the sweet taste of the hydrogenated stevioside (**6**/**7**) and rebaudioside-A (**8**/**9**) compounds was reduced by about 50%, whereas the rubusoside derivatives (**4**/**5**) completely lost their sweetness after catalytic hydrogenation (Table 1). These results indicated that the C16-C17 methylene double bond in steviol glycosides can be regarded as a pharmacophore essential for the sweetness property of these molecules [14,15].

### 2.2. Spectroscopy

The structural characterization of **4**–**9** were performed on the basis of one dimensional (^1^H, ^13^C), two-dimensional (^1^H-^1^H COSY, ^1^H-^13^C HMQC, ^1^H-^13^C HMBC) NMR and mass spectral data. The stereochemistry at the C-16 position was identified by comparison with their corresponding aglycone derivative literature NMR values [16,17,18], as well as enzymatic hydrolysis studies. The ^1^H- and ^13^C-NMR values for all the protons and carbons in **4**–**9** were assigned on the basis of COSY, HMQC and HMBC correlations. Further it was found that the ratio of 17α/17β reduced compounds were observed at 3:2 for compounds rubusoside (**1**) and rebaudioside (**3**), whereas it was 1:1 in case of stevioside (**2**). The ^1^H-NMR data for the key protons in **4**–**9** were given in Table 2, whereas the complete assignments of their carbon values were given in Table 3. 

**Table 1:** Sensory evaluation of rubusoside (**1**), stevioside (**2**) and rebaudioside-A (**3**) verses catalytically hydrogenated steviol glycosides (**4**–**9**) at 500 ppm in CT water at rt.

Steviol Glycoside Type	Sensory Evaluation of Original Compound	Sensory Evaluation of Reduced Compound
Rubusoside	Slow onset of sweetness, about 2–3% sucrose equivalence	No sweetness
Stevioside	Slow onset of sweetness, sweet lingering aftertaste, about 5–6% sucrose equivalence	Slow onset of sweetness, less sweetness linger to original, about 2–3% sucrose equivalence
Rebaudioside A	Slow onset of sweetness, sweet lingering aftertaste, about 6–7% sucrose equivalence	Slow onset of sweetness, less sweetness linger to original, about 3–4% sucrose equivalence

**Table 2:** ^1^H-NMR chemical shifts values for reduced compounds **4**–**9** recorded in C_5_D_5_N ^a-c^.

Position	4	5	6	7	8	9
17	1.10 (d, 6.4, 1H)	1.16 (d, 6.5, 1H)	1.21 (d, 6.7, 1H)	1.28 (d, 6.4, 1H)	1.11 (d, 6.5, 1H)	1.17 (d, 6.5, 1H)
18	1.25 (s, 3H)	1.25 (s, 3H)	1.25 (s, 3H)	1.25 (s, 3H)	1.25 (s, 3H)	1.25 (s, 3H)
20	1.32 (s, 3H)	1.32 (s, 3H)	1.29 (s, 3H)	1.29 (s, 3H)	1.31 (s, 3H)	1.31 (s, 3H)
1′	6.16 (d, 6.8, 1H)	6.14 (d, 6.6, 1H)	6.15 (d, 6.8, 1H)	6.15 (d, 6.5, 1H)	6.15 (d, 6.5, 1H)	6.17 (d, 6.5, 1H)
1′′	5.03 (d, 6.7, 1H)	5.02 (d, 6.6, 1H)	5.09 (d, 6.7, 1H)	5.04 (d, 6.9, 1H)	5.00 (d, 6.7, 1H)	5.03 (d, 6.9, 1H)
1′′′			5.25 (d, 6.5, 1H)	5.27 (d, 6.8, 1H)	5.35 (d, 6.6, 1H)	5.32 (d, 6.5, 1H)
1′′′′					5.52 (d, 6.4, 1H)	5.44 (d, 6.8, 1H)

^a^ assignments made on the basis of COSY, HMQC and HMBC correlations; ^b^ Chemical shift values are in δ (ppm); ^c^ Coupling constants are in Hz.

**Table 3:** ^13^C-NMR chemical shifts values for reduced compounds **4**–**9** recorded in C_5_D_5_N ^a-b^.

Position	4	5	6	7	8	9
1	41.3	41.3	41.2	41.2	41.2	41.2
2	20.4	20.3	20.1	20.1	20.1	20.1
3	38.9	38.8	38.9	38.8	38.9	38.8
4	45.1	43.0	44.8	43.1	44.9	43.0
5	57.9	57.9	57.8	57.8	57.8	57.8
6	22.8	23.2	22.7	23.0	22.8	23.0
7	41.5	40.3	41.7	40.1	41.7	40.2
8	44.1	43.1	44.3	43.0	44.4	43.1
9	55.9	54.9	55.6	54.6	55.6	54.7
10	40.2	40.3	40.1	40.2	40.1	40.3
11	20.5	20.8	20.3	20.6	20.4	20.6
12	36.6	44.1	35.3	44.2	35.4	44.3
13	86.0	85.8	86.2	86.2	88.2	88.1
14	47.5	50.8	47.3	50.6	47.2	50.6
15	47.5	44.6	47.2	44.9	47.3	44.8
16	41.3	38.8	41.2	39.0	41.2	38.9
17	14.2	19.9	14.2	19.7	14.4	19.8
18	28.6	28.7	28.6	28.6	28.6	28.6
19	177.5	177.6	177.4	177.5	177.5	177.6
20	15.9	16.1	15.8	16.0	15.7	15.9
1′	96.3	96.3	96.2	96.2	96.2	96.2
2′	75.9	75.9	74.3	74.4	75.7	75.6
3′	79.6	79.7	79.6	79.7	79.6	79.8
4′	71.6	71.5	71.4	71.4	71.5	71.4
5′	78.3	78.5	78.2	78.4	78.5	78.6
6′	63.7	63.8	63.1	63.4	63.1	63.3
1′′	100.4	99.8	98.3	98.4	99.0	98.8
2′′	74.5	74.5	84.3	84.7	78.4	78.5
3′′	79.3	79.3	78.1	78.1	86.5	85.6
4′′	73.0	73.2	72.9	72.9	71.9	72.0
5′′	79.7	79.8	79.5	79.5	77.2	77.0
6′′	62.5	62.5	62.7	62.7	62.7	62.8
1′′′			106.8	107.1	105.1	105.5
2′′′			74.3	74.3	74.6	74.6
3′′′			77.5	77.7	77.6	77.8
4′′′			71.7	71.9	71.9	72.0
5′′′			79.2	79.2	78.9	79.0
6′′′			62.4	62.4	62.5	62.5
1′′′′					105.3	105.9
2′′′′					74.3	74.4
3′′′′					79.8	79.9
4′′′′					72.2	72.1
5′′′′					79.6	79.7
6′′′′					63.2	63.1

^a^ assignments made on the basis of COSY, HMQC and HMBC correlations; ^b^ Chemical shift values are in δ (ppm).

## 3. Experimental

### 3.1. General

Melting points were measured using a SRS Optimelt MPA 100 instrument and are uncorrected. IR spectral data was acquired using a Perkin Elmer 400 Fourier Transform Infrared (FT-IR) Spectrometer (Atlanta, USA) equipped with a Universal Attenuated Total Reflectance (UATR, Atlanta, USA) polarization accessory, whereas NMR spectra were acquired on Varian Unity Plus 600 MHz instrument (Atlanta, USA) in C_5_D_5_N using standard pulse sequences. Chemical shifts were given in δ (ppm), and coupling constants were reported in Hz. HRMS and MS/MS data were generated with a Waters Premier Quadrupole Time-of-Flight (Q-TOF, New Jersey, USA) mass spectrometer equipped with an electrospray ionization source operated in the positive-ion mode and ThermoFisher Discovery OrbiTrap (New Jersey, USA) in the positive mode of electrospray. All the samples were diluted with water: acetonitrile (1:1) containing 0.1% formic acid and introduced via infusion using the onboard syringe pump. CT water was prepared by passing water through granular or block carbon material to reduce toxic compounds as well as harmless taste- and odor-producing chemicals. 

### 3.2. Isolation of Reduced Steviol Glycosides ***4**–**9***

#### 3.2.1. General Procedure for the Catalytic Hydrogenation of Steviol Glycosides **1**–**3**

To a solution of each steviol glycoside **1***–***3** (2 g) in EtOH/H_2_O (8:2, 100 mL) was added Pd(OH)_2_ (50 mg). The mixture was hydrogenated at ambient temperature for 5 days under 55 psi H_2_. After each day an aliquot of the sample was filtered through Celite and analyzed by HPLC for the absence of starting materials. At the end of hydrogenation (5 days), the reaction mixture was filtered through celite and concentrated under vacuum to afford product until a clear white product was formed. The product was triturated in acetone and filtered and dried under vacuum at 50 °C for 2 days. The combined purity of each isomeric mixture **4**/**5**, **6**/**7**, and **8**/**9** was checked by HPLC and was found >99%.

*Dihydrorubusoside 1/Dihydrorubusoside 2* (**4**/**5**). White powder; IR ν_max_: 3352, 2924, 2880, 1725, 1032, 893 cm^−1^; ^1^H-NMR and ^13^C-NMR spectroscopic data see Table 2 and Table 3 respectively; HRMS (M+NH_4_)^+^*m/z* 662.3752 (calcd. for C_32_H_56_NO_13_: 662.3753), (M+Na)^+^*m/z* 667.3306 (calcd. for C_32_H_52_O_13_Na: 667.3306).

*Dihydrostevioside 1/Dihydrostevioside 2* (**6**/**7**). White powder; IR ν_max_: 3345, 2926, 2883, 1728, 1035, 895 cm^−1^; ^1^H-NMR and ^13^C-NMR spectroscopic data see Table 2 and Table 3 respectively; HRMS (M+H)^+^*m/z* 807.4021 (calcd. for C_38_H_63_O_18_: 807.4014), (M+NH_4_)^+^*m/z* 824.4284 (calcd. for C_38_H_66_NO_18_: 824.4280).

*Dihydrorebaudiososide-A1/Dihydrorebaudiososide-A2* (**8**/**9**). White powder; IR ν_max_: 3347, 2923, 2885, 1732, 1030, 885 cm^−1^; ^1^H-NMR and ^13^C-NMR spectroscopic data see Table 2 and Table 3 respectively; HRMS (M+H)^+^*m/z* 969.4553 (calcd. for C_44_H_73_O_23_: 969.4543), (M+NH_4_)^+^*m/z* 986.4808 (calcd. for C_44_H_76_NO_23_: 986.4808).

#### 3.2.2. General Procedure for the Enzymatic Hydrolysis of Reduced Steviol Glycoside Mixtures

**Figure 2:**
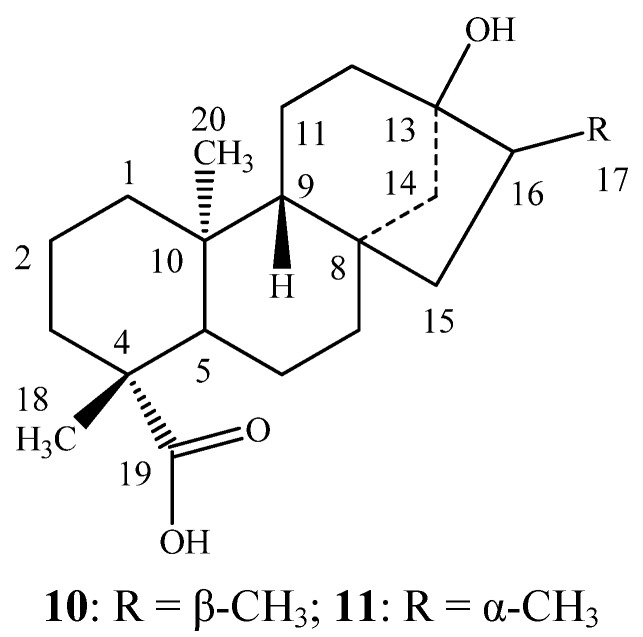
Structures of dihydrosteviol A (**10**) and dihydrosteviol B (**11**).

The mixture of each reduced steviol glycoside (100 mg) was dissolved in 0.1 M sodium acetate buffer, pH 4.5 (25 mL) and crude pectinase from *Aspergillus niger* (5 mL, Sigma-Aldrich, P2736) was added. The mixture was stirred at 50 °C for 96 h. The product precipitated out during the reaction for all three mixtures **4**/**5**, **6**/**7** and **8**/**9** was identified as the same. The filtered compound was purified over silica gel column chromatography; elution with *n*-hexane/acetone (9.5:0.5) yielded dihydrosteviol A (**10**, 8 mg, m.p.: 189–192 °C) whereas elution with *n*-hexane/acetone (9.0:1.0) yielded dihydrosteviol B (**11**, 6 mg, m.p.: 214–217 °C). The two compounds **10**–**11** (Figure 2) were identified by comparison of their physical and ^1^H-NMR spectral data with the literature values [16,17,18]. 

### 3.3. Sensory Evaluation of the Reduced Steviol Glycoside Mixtures

Sweetness evaluation of the reduced steviol glycoside mixtures was performed using sucrose as a control along with their original compounds. The extra fine cane sucrose from Domino (lot#11:09 6843 1A10) was used for preparation. The reduced steviol glycoside mixtures at 500 ppm for evaluation were prepared by adding a non-moisture compensated mass into a 100 mL sample of carbon-treated (CT) water. The mixtures were moderately stirred at room temperature (rt) and the reduced steviol glycoside samples were then evaluated against several control sucrose samples at 0.75%, 2%, 4%, 6% and 7.0% SE in water at RT by experienced Research and Technology panelists at The Coca-Cola Company, Atlanta, USA, for any tasting quality determinations using the controlled, multi-sip and swallow taste method shown below:

### 3.4. Multi-Sip and Swallow Taste Method

1. Take 1st sip (~1.8 mL) of a full medicine cup and swallow the control, wait for 15–25 s, then take the 2nd sip and lock it into memory and wait for 15–25 s.

2. Taste the 1st sip of the experimental sample; wait for 15–25 s, then use the 2nd sip to compare to the 2nd of the sip control.

3. Then repeat steps #1 and #2 for the 3rd and 4th sips of the same control and experimental samples just to confirm the initial finding.

## 4. Conclusions

In conclusion, six *ent*-kaurane diterpene glycosides 4–9 were synthesized from the natural products rubusoside, stevioside, and rebaudioside-A by hydrogenation carried out using Pd(OH)_2_ as the catalyst. The structures of all synthesized compounds were characterized on the basis of NMR (1D and 2D) and mass spectral data, enzymatic hydrolysis as well as in comparison with the data reported in the literature. To the best of our knowledge, this is the first report of the complete spectral characterization of the reduced compounds of rubusoside, stevioside, and rebaudioside-A. 
